# The Eating Motivation Survey in Brazil: Results From a Sample of the General Adult Population

**DOI:** 10.3389/fpsyg.2019.02334

**Published:** 2019-10-15

**Authors:** Gudrun Sproesser, Jéssica Maria Muniz Moraes, Britta Renner, Marle dos Santos Alvarenga

**Affiliations:** ^1^Psychological Assessment and Health Psychology, University of Konstanz, Konstanz, Germany; ^2^Department of Nutrition, School of Public Health, University of São Paulo, São Paulo, Brazil

**Keywords:** The Eating Motivation Survey, cross-country replication, consistent structure, food choice motives, Brazil, cross-cultural validity

## Abstract

Brazil is undergoing a rapid nutrition transition with unfavorable effects on dietary healthiness. To counteract this trend, knowledge about individual drivers of consumption is needed that go beyond environmental factors. The Eating Motivation Survey represents a comprehensive measure of such individual drivers, assessing 15 eating motives, such as choosing food for health reasons or eating because of a good taste. The aim of this study was to examine the psychometric properties and validity of a Brazilian Portuguese version of The Eating Motivation Survey. In total, 442 participants from the general adult population living in the state of São Paulo, Brazil filled in an online survey. Results showed that the model, including 15 motive factors and 45 items, had a reasonable model fit. Moreover, factor loadings and corrected item-scale correlations were generally good. Fourteen out of the 15 motive scales had a reliability above 0.60. Results also confirmed convergent validity. These results demonstrate that the Brazilian Portuguese version of The Eating Motivation Survey is generally reliable and valid to assess individual drivers of eating behavior in Brazil.

## Introduction

Within the past few decades, Brazil has experienced a more than fourfold increase in obesity rates. According to the World Health Organization (World Health Organization [WHO], 2019), obesity prevalence in adults increased from 5.2% in 1975 to 22.1% in 2016. This increase in obesity prevalence is accompanied both by an increase in non-communicable diseases (Marinho et al., 2018) and a nutrition transition (Monteiro et al., 2004; Rodrigues et al., 2016). For instance, the consumption of ultra-processed foods, which are relatively high in added sugar, saturated fat, sodium, and energy, has increased in Brazil from 18.7% of total calories purchased in 1987 to 29.6% in 2009 (Martins et al., 2013). To slow down these trends, an understanding of the drivers of eating behavior is crucial. There are many environmental determinants of eating behavior, such as access to new technologies, modern supermarkets, or food marketing (Kearney, 2010; Popkin et al., 2012). For instance, the technology of refrigeration made it possible to lengthen the season of foods (Root and de Rochemont, 1981). However, environmental factors cannot explain why, in similar environments, people still differ in their eating practices. Therefore, individual factors need to be examined.

One approach concerning individual drivers of eating behavior is the investigation of eating motives, that is, why people eat what they eat. The first systematic attempt to assess the different motives for why people eat what they eat was the development of the Food Choice Questionnaire that includes nine motive scales (FCQ; Steptoe et al., 1995). However, as important motives such as social or physiological motives were not included in the FCQ, The Eating Motivation Survey (TEMS) was developed encompassing fifteen basic motives (Renner et al., 2012). These motives are good taste, habit, hunger, health concerns, convenience, pleasure, traditions, natural concerns, sociability, price considerations, the visual appeal of foods, weight control concerns, to regulate negative affect, social norms, and social image concerns. The fifteen motives have consistently been found across gender, age, and BMI groups (Renner et al., 2012; Rempe et al., 2018). Yet, this consistency does not mean that there are no group differences in the mean level of motives. For instance, in samples from France, Germany, the Netherlands, Poland, and Spain, people with a higher BMI displayed significantly higher weight control concerns, eating to regulate negative affect, and to comply with social norms (Renner et al., 2012; van Strien et al., 2016), whereas people with a lower BMI had higher values for eating because of good taste, hunger, and health concerns (Pieniak et al., 2009a; Renner et al., 2012).

TEMS has been applied in different countries, with satisfying results regarding its reliability and validity. Specifically, TEMS has been applied in Germany (Renner et al., 2012), the United States (Arbit et al., 2017; Phan and Chambers IV, 2018), Portugal (Graça et al., 2019), Finland (Vainio, 2019), China (Siegrist et al., 2015), the United Kingdom (Pechey et al., 2015), Turkey (Chambers et al., 2016; Avsar et al., 2017), and Australia (Skead et al., 2018). In a sample of Indians, US Americans and Germans, Sproesser et al. (2018) confirmed the 15-factor structure of TEMS across countries, whereas samples of the three countries differed in the mean levels of motives. Recently, a Brazilian Portuguese version has been developed (Moraes and Alvarenga, 2017). However, to the best of authors’ knowledge, there has not yet been an investigation of the psychometric properties and validity of this Brazilian Portuguese version of TEMS. Hence, the first aim of this study was to examine the factor structure of the Brazilian Portuguese TEMS in confirmatory factor analysis and to investigate its scale reliabilities. Second, this study aimed to investigate the construct validity of TEMS by relating the 15 TEMS motives to the nine motives of the FCQ as well as to investigate the relationship of TEMS scales with BMI.

## Materials and Methods

### Participants

In total, 681 participants consented to take part in the study. Out of these, 239 participants (35%) filled out less than 75% of the survey and were therefore excluded from analysis (please see section Analytical Procedure). The remaining 442 participants (366 women, 83%) had a mean age of 39.5 years (*SD* = 12.8; *Md* = 38 years; range 18–77 years). The average BMI was 25.8 kg/m^2^ (*SD* = 5.3; range 15.1–45.8 kg/m^2^) and the mean education level was 5.9 (*SD* = 1.2; range 1 – 8) as classified by the International Standard Classification of Education 2011 (OECD, 2015) with 88% indicating tertiary education, 11% upper secondary education, and 1% below upper secondary education. As a further data quality check, the duration to complete the survey was inspected for potential ‘speeders,’ that is people who speeded through the survey with random responding. Of the 442 participants, none had a z-standardized completion duration below −3. Hence, none of the 442 participants had to be excluded due to ‘speeding.’ The sample size meets the suggestion for a minimum of 200 participants for confirmatory factor analyses (Barrett, 2007).

Compared with Brazilian population data, the sample comprised of more females (51% in Brazilian population, United Nations, 2019), was older (*Md* = 31.4 years in Brazilian population in 2015; United Nations, 2019), had a lower BMI (*M* = 26.6 kg/m^2^ in Brazilian population in 2016; World Health Organization [WHO], 2019), and was better educated (in 2015, 15% of the Brazilian population had tertiary education, 34% had upper secondary education, and 51% had below upper secondary education; OECD, 2019).

Comparing the study sample to the drop-out sample showed no significant differences in terms of gender [83% vs. 88% females, χ^2^(1) = 2.66, *p* = 0.103], BMI [25.8 kg/m^2^ vs. 25.6 kg/m^2^, *t*(481) = 0.36, *p* = 0.723], and education level [5.9 vs. 5.7, *t*(339.92) = 1.75, *p* = 0.081]. However, the study sample was slightly younger [39.5 years vs. 42.1 years, *t*(310.84) = −1.98, *p* = 0.049] than the drop-out sample.

### Measures

#### Demographics

Participants were asked to indicate their gender, age, height, weight, and education level. Age, height, and weight were assessed via a free text response; gender via a closed-ended format. People’s highest level of education was also assessed via a closed-ended format and categorized in line with the International Standard Classification of Education 2011 (OECD, 2015) as 0 ‘Early childhood education,’ 1 ‘Primary education,’ 2 ‘Lower secondary education,’ 3 ‘Upper secondary education,’ 4 ‘Post-secondary non-tertiary education,’ 5 ‘Short-cycle tertiary education,’ 6 ‘Bachelor’s or equivalent level,’ 7 ‘Master’s or equivalent level,’ 8 ‘Doctoral or equivalent level.’

#### Eating Motives

Participants completed a Brazilian Portuguese version of the brief Eating Motivation Survey (Renner et al., 2012; Moraes and Alvarenga, 2017; Sproesser et al., 2018). The translation into Brazilian Portuguese was done following guidelines for cross-cultural adaptation processes (Beaton et al., 2000; Reichenheim and Moraes, 2007). This process included the following stages (see also Moraes and Alvarenga, 2017):

(1) Conceptual and item equivalence: This stage consisted of exploring the construct of interest (eating motivations) and the weights given to its different constituent domains in Brazil in a discussion with a group of specialists with scientific expertise regarding eating motivations in Brazil. This discussion targeted the question whether the various domains covered by TEMS are relevant and pertinent to assess the concepts of interest in Brazil. The pertinence of each TEMS item for assessing the domains was evaluated.
(2) Semantic equivalence: In this stage, TEMS was translated into Portuguese with special attention that the meaning of items and concepts was maintained. Specifically, the English TEMS was first translated into Brazilian Portuguese by two translators. The two translations were discussed by two of the authors of this study (JM and MA) and collapsed into a first translation version. Next, the items of this first version were evaluated by 22 experts in the domain of eating behavior with regard to their understandability. Items with suboptimal understandability were revised by one linguist, resulting in a second translation version. This translation version as well as the English TEMS were filled in by 23 bilinguals and responses to the English and Portuguese items were compared. Items with substantial differences between the English and Portuguese version were revised, resulting in a third translation version. This third version was back-translated into English; differences between the original and back-translation were solved by discussion between three of the authors (GS, MA, and JM).
(3) Operational equivalence: In this stage, attention was paid toward the application of the translated version being equivalent to the original version. Specifically, this included a pretest of the Brazilian Portuguese version with 32 participants. In line with the participants’ comments, the response format was changed from the original 7 to 5 categories from 1 ‘never’ to 5 ‘always.’ The Brazilian Portuguese items are displayed in the Supplementary Table S1.

To investigate construct validity, participants also completed the Brazilian Portuguese version of the Food Choice Questionnaire (FCQ; Steptoe et al., 1995; Heitor et al., 2015). The FCQ consists of the nine food choice motive scales Health (α = 0.85), Mood (α = 0.86), Convenience (α = 0.88), Sensory Appeal (α = 0.78), Natural Content (α = 0.88), Price (α = 0.82), Weight Control (α = 0.87), Familiarity (α = 0.76), and Ethical Concern (α = 0.80). The 36 items are preceded by the item stem ‘It is important to me that the food I eat on a typical day …’ and responses are given on a 4-point rating scale from 1 ‘not at all important’ to 4 ‘very important.’

### Procedure

Data were collected in an online survey study with a convenience sample living in the state of São Paulo (Qualtrics survey software). The study was announced as a study investigating eating motivations in Brazil, that is why people eat what they eat. Participants were recruited via the snowball technique, advertising the study to Facebook members, and via an online panel company. Within the snowball technique, participants were invited to the study through e-mails sent to e-mail groups and individual contacts. Participants were free to forward the link to their acquaintances in order to recruit additional participants. In Facebook, the advertisement was shown to Facebook members of 18 years or older, living in the state of São Paulo. Participants were excluded from the study if they did not provide informed consent or were younger than 18 years. Besides that, there were no further exclusion or inclusion criteria. Participants who were recruited via the snowballing technique and via Facebook advertising did not receive an incentive. Participants who were recruited via the online panel company were remunerated by the company. The ethics board of the Public Health School from the University of São Paulo approved the study protocol. The procedures were performed in compliance with relevant laws and institutional guidelines. We followed the German Psychological Society’s (Deutsche Gesellschaft für Psychologie) guidelines for conducting psychological studies^1^. These are similar to those of the American Psychological Association. The study conforms to the Declaration of Helsinki. All participants consented to participate in this study after being fully informed about the study.

### Analytical Procedure

Statistical analyses were conducted using IBM SPSS and AMOS (Versions 25 for Windows). As complete case analyses have been criticized as not being representative for the whole sample (e.g., Graham, 2012), missing data in TEMS and the FCQ were imputed using the Expectation Maximization algorithm in SPSS (Gold and Bentler, 2000). To ensure that missing values were below 5% for all imputed variables (a recommendation for using the Expectation Maximization algorithm; Schafer, 1999), participants who filled in less than 75% of the survey were excluded (please see Section Participants). Skewness and excess of all TEMS items were below the thresholds of 2 and 7, respectively, as suggested by Curran et al. (1996).

Confirmatory factor analyses (CFAs) using maximum likelihood solutions were conducted. The item with the highest factor loading was fixed to 1.0 for each factor, respectively. Model fit was assessed by the comparative fit index (CFI), the standardized root mean squared residual (SRMR), and the root-mean-square error of approximation (RMSEA) as recommended by Kline (2011). A reasonable fit is indicated by a CFI ≥ 0.90, a SRMR value ≤ 0.10, and a RMSEA value ≤ 0.08 (Kline, 2011). Because the χ^2^ statistic is sample-size dependent, the χ^2^/*df* ratio was additionally calculated with a χ^2^ not larger than 2–5 times the degrees of freedom indicating a good fit (Bollen and Long, 1993).

## Results

Means, standard deviations, standardized factor loadings, and corrected item-scale correlations of the 45 items are displayed in Table 1. Motive correlations and internal consistencies are listed in Table 2. All factor loadings were statistically significant (*p* < 0.001) and above the recommended level of 0.30 (Kline, 2011). Motive correlations ranged from −0.26 (‘Liking’ and ‘Weight Control’) to 0.57 (‘Health’ and ‘Natural Concerns’). Reliabilities were good for 11 out of the 15 motive scales with values higher than 0.70. Only one out of the 15 motive scales had a reliability lower than 0.60, namely the scale ‘Need and Hunger.’ Within this scale, two items had corrected item-scale correlations lower than 0.30 (‘… because I need energy’ and ‘… because I am hungry’). The remaining 43 items showed corrected item-scale correlations higher than 0.30.

**TABLE 1:** Means (M), standard deviations (SD), standardized factor loadings (a), and corrected item-scale correlations [*r*_*i(t–i*__)_] for TEMS items in confirmatory factor analysis (*N* = 442).

**I eat what I eat…**	***M***	***SD***	***a***	***r*_*i(t–i)*_**
**Liking**	3.91	0.66		
… because I have an appetite for it.	3.71	0.86	0.66	0.56
… because it tastes good.	3.88	0.80	0.83	0.68
… because I like it.	4.14	0.73	0.69	0.55
**Habits**	3.60	0.69		
… because I am accustomed to eating it.	3.50	0.89	0.77	0.57
… because I usually eat it.	3.52	0.87	0.75	0.59
… because I am familiar with it.	3.76	0.85	0.49	0.40
**Need and Hunger**	3.60	0.62		
… because I need energy.	3.10	1.05	0.34	0.21
… because it is pleasantly filling.	3.82	0.86	0.65	0.35
… because I’m hungry.	3.88	0.79	0.45	0.25
**Health**	3.34	0.81		
… to maintain a balanced diet.	3.44	0.96	0.84	0.68
… because it is healthy.	3.46	0.97	0.84	0.64
… because it keeps me in shape (e.g., energetic, motivated).	3.13	1.05	0.50	0.45
**Convenience**	3.04	0.80		
… because it is quick to prepare.	3.08	0.91	0.75	0.66
… because it is convenient.	2.95	1.01	0.68	0.58
… because it is easy to prepare.	3.08	0.93	0.85	0.70
**Pleasure**	3.09	0.77		
… because I enjoy it.	3.83	0.88	0.52	0.38
… in order to indulge myself.	2.97	0.99	0.73	0.62
… in order to reward myself.	2.48	1.06	0.72	0.50
**Traditional Eating**	2.92	0.75		
… because it belongs to certain situations.	2.94	0.96	0.60	0.38
… out of traditions (e.g., family traditions, special occasions).	2.94	0.99	0.73	0.53
… because I grew up with it.	2.87	1.07	0.46	0.33
**Natural Concerns**	2.86	0.84		
… because it is natural.	3.25	1.04	0.60	0.45
… because it contains no harmful substances (e.g., pesticides, pollutants, antibiotics).	2.92	1.10	0.74	0.56
… because it stems from organic farming.	2.42	1.02	0.73	0.62
**Sociability**	2.95	0.86		
… because it is social.	2.87	1.04	0.68	0.57
… so that I can spend time with other people.	2.83	0.98	0.76	0.64
… because it makes social gatherings more comfortable.	3.14	1.06	0.80	0.68
**Price**	2.67	0.85		
… because it is inexpensive.	2.64	0.99	0.79	0.68
… because I don’t want to spend any more money.	2.67	1.00	0.80	0.71
… because it is on sale.	2.70	0.98	0.76	0.66
**Visual Appeal**	2.36	0.80		
… because the presentation is appealing (e.g., packaging).	2.79	1.03	0.64	0.58
… because it spontaneously appeals to me (e.g., situated at eye level, appealing colors).	2.29	0.97	0.82	0.71
… because I recognize it from advertisements or have seen it on TV.	1.99	0.89	0.75	0.56
**Weight Control**	2.52	0.92		
… because it is low in calories.	2.36	1.04	0.78	0.69
… because I watch my weight.	2.63	1.12	0.76	0.64
… because it is low in fat.	2.58	1.06	0.76	0.64
**Affect Regulation**	2.14	0.99		
… because I am sad.	2.22	1.07	0.92	0.85
… because I am frustrated.	2.26	1.10	0.94	0.87
… because I feel lonely.	1.94	1.05	0.79	0.76
**Social Norms**	2.18	0.77		
… because it would be impolite not to eat it.	2.02	0.89	0.82	0.56
… to avoid disappointing someone who is trying to make me happy.	2.15	0.94	0.81	0.60
… because I am supposed to eat it.	2.37	1.12	0.40	0.34
**Social Image**	1.56	0.67		
… because it is trendy.	1.55	0.76	0.76	0.68
… because it makes me look good in front of others.	1.56	0.80	0.78	0.68
… because others like it.	1.55	0.78	0.81	0.69

**TABLE 2:** Pearson correlations between motives of The Eating Motivation Survey and internal consistencies (Cronbach’s alphas; *N* = 442).

	**1**	**2**	**3**	**4**	**5**	**6**	**7**	**8**	**9**	**10**	**11**	**12**	**13**	**14**	**15**
(1) Liking	1														
(2) Habits	0.24^∗∗∗^	1													
(3) Hunger	0.31^∗∗∗^	0.32^∗∗∗^	1												
(4) Health	–0.04	0.28^∗∗∗^	0.43^∗∗∗^	1											
(5) Convenience	0.13^∗∗^	0.13^∗∗^	0.17^∗∗^	−0.09^∗^	1										
(6) Pleasure	0.42^∗∗∗^	0.15^∗∗^	0.23^∗∗∗^	0.03	0.32^∗∗∗^	1									
(7) Traditional Eating	0.25^∗∗∗^	0.27^∗∗∗^	0.21^∗∗∗^	0.08	0.22^∗∗∗^	0.35^∗∗∗^	1								
(8) Natural Concerns	–0.02	0.25^∗∗∗^	0.23^∗∗∗^	0.57^∗∗∗^	–0.03	0.10^∗^	0.08	1							
(9) Sociability	0.23^∗∗∗^	0.18^∗∗∗^	0.26^∗∗∗^	0.15^∗∗^	0.25^∗∗∗^	0.42^∗∗∗^	0.54^∗∗∗^	0.18^∗∗∗^	1						
(10) Price	0.10^∗^	0.12^∗^	0.16^∗∗^	−0.11^∗^	0.50^∗∗∗^	0.23^∗∗∗^	0.15^∗∗^	–0.06	0.25^∗∗∗^	1					
(11) Visual Appeal	0.15^∗∗^	0.04	0.06	–0.14^∗∗^	0.38^∗∗∗^	0.28^∗∗∗^	0.29^∗∗∗^	–0.04	0.33^∗∗∗^	0.42^∗∗∗^	1				
(12) Weight Control	–0.26^∗∗∗^	0.13^∗∗^	0.04	0.43^∗∗∗^	0.10^∗^	0.01	–0.02	0.33^∗∗∗^	0.04	0.05	0.09	1			
(13) Affect Regulation	0.18^∗∗∗^	0.10^∗^	–0.05	–0.07	0.25^∗∗∗^	0.46^∗∗∗^	0.30^∗∗∗^	0.03	0.35^∗∗∗^	0.21^∗∗∗^	0.28^∗∗∗^	0.15^∗∗^	1		
(14) Social Norms	0.02	0.10^∗^	0.10^∗^	–0.02	0.31^∗∗∗^	0.23^∗∗∗^	0.35^∗∗∗^	0.05	0.43^∗∗∗^	0.31^∗∗∗^	0.33^∗∗∗^	0.14^∗∗^	0.40^∗∗∗^	1	
(15) Social Image	–0.02	0.07	0.00	0.05	0.25^∗∗∗^	0.21^∗∗∗^	0.24^∗∗∗^	0.12^∗∗^	0.30^∗∗∗^	0.30^∗∗∗^	0.44^∗∗∗^	0.27^∗∗∗^	0.35^∗∗∗^	0.47^∗∗∗^	1
α	0.76	0.70	0.44	0.75	0.80	0.68	0.60	0.72	0.79	0.83	0.78	0.81	0.92	0.67	0.83

*^∗^*p* < 0.05; ^∗∗^*p* < 0.01; ^∗∗∗^*p* < 0.001.*

Model fit was generally reasonable. The Chi-square statistic was 2207 with df = 840 and *p* < 0.001, indicating no exact fit of the model, which is to be expected considering the large sample sizes (Kline, 2011). Despite the complexity of the model with 15 factors and 45 items, the χ^2^/df ratio was 2.63, indicating a good approximate model fit. Also, the SRMR and RMSEA were 0.070 and 0.061 (90% CI: 0.058 − 0.064) respectively, demonstrating a reasonable approximate model fit. Only the CFI was 0.85 and hence below the recommended threshold of 0.90. However, according to Heene et al. (2011) the CFI needs to be interpreted with caution when sample sizes are below *N* = 500.

To investigate construct validity, TEMS scales were correlated with the scales of the Food Choice Questionnaire (Steptoe et al., 1995; Heitor et al., 2015; see Table 3). Within each FCQ scale, highest correlations were observed with the respective TEMS scale(s) that taps into a similar construct, indicating convergent validity. These correlations are set in bold in Table 3. For instance, the FCQ scale ‘Sensory Appeal’ taps into choosing foods because they taste or look good. Similarly, the TEMS scales ‘Liking’ and ‘Visual Appeal’ measure eating because of good taste and appealing presentation. The FCQ scale ‘Sensory Appeal’ correlated highest with the TEMS scales ‘Liking’ (*r* = 0.31) and ‘Visual Appeal’ (*r* = 0.23). Similarly, the FCQ scale ‘Weight Control’ displayed the highest correlation with the TEMS scale ‘Weight Control’ (*r* = 0.72), as was the case for the FCQ and TEMS scales ‘Price’ (*r* = 0.54), FCQ and TEMS scales ‘Convenience’ (*r* = 0.56), as well as FCQ and TEMS scales ‘Health’ (*r* = 0.57).

**TABLE 3:** Pearson correlations between scales of The Eating Motivation Survey (TEMS) and scales of the Food Choice Questionnaire (FCQ) and BMI (*N* = 442).

	**FCQ scales**									**BMI**
**TEMS scales**	**Sensory Appeal**	**Familiarity**	**Health**	**Convenience**	**Mood**	**Natutal Content**	**Ethical Concerns**	**Price**	**Weight Control**	
Liking	**0.31^∗∗∗^**	0.04	–0.13^∗∗^	0.03	0.11^∗^	–0.04	–0.06	0.06	–0.23^∗∗∗^	–0.07
Habits	0.18^∗∗∗^	**0.24^∗∗∗^**	0.13^∗∗^	0.03	0.13^∗∗^	0.11^∗^	0.12^∗^	0.07	–0.01	–0.04
Hunger	0.23^∗∗∗^	0.16^∗∗^	0.20^∗∗∗^	0.13^∗∗^	0.19^∗∗∗^	0.14^∗∗^	0.09	0.15^∗∗^	0.02	–0.18^∗∗∗^
Health	0.12^∗^	0.12^∗∗^	**0.57^∗∗∗^**	0.00	0.18^∗∗∗^	0.52^∗∗∗^	0.40^∗∗∗^	0.09	0.25^∗∗∗^	–0.18^∗∗∗^
Convenience	0.15^∗∗^	0.11^∗^	0.02	**0.56^∗∗∗^**	0.31^∗∗∗^	–0.06	–0.04	0.29^∗∗∗^	0.17^∗∗∗^	0.14^∗∗^
Pleasure	0.17^∗∗∗^	0.04	–0.08	0.13^∗∗^	**0.36^∗∗∗^**	–0.08	–0.02	0.06	–0.05	0.10^∗^
Traditional Eating	0.17^∗∗∗^	**0.39^∗∗∗^**	–0.06	0.09	0.13^∗∗^	–0.03	0.01	0.08	–0.06	0.08
Natural Concerns	0.01	0.11^∗^	0.45^∗∗∗^	0.01	0.22^∗∗∗^	**0.64^∗∗∗^**	**0.57^∗∗∗^**	0.11^∗^	0.22^∗∗∗^	–0.05
Sociability	0.22^∗∗∗^	0.21^∗∗∗^	0.07	0.19^∗∗∗^	0.29^∗∗∗^	0.07	0.05	0.10^∗^	–0.03	0.05
Price	0.05	0.13^∗∗^	0.11^∗^	0.40^∗∗∗^	0.29^∗∗∗^	–0.06	–0.00	**0.54^∗∗∗^**	0.14^∗∗^	0.15^∗∗^
Visual Appeal	**0.23^∗∗∗^**	0.26^∗∗∗^	0.01	0.27^∗∗∗^	0.25^∗∗∗^	−0.10^∗^	–0.02	0.29^∗∗∗^	0.13^∗∗^	0.16^∗∗^
Weight Control	0.01	0.04	0.50^∗∗∗^	0.14^∗∗^	0.23^∗∗∗^	0.32^∗∗∗^	0.26^∗∗∗^	0.14^∗∗^	**0.72^∗∗∗^**	0.16^∗∗^
Affect Regulation	0.06	0.10^∗^	–0.05	0.20^∗∗∗^	**0.39^∗∗∗^**	–0.06	–0.01	0.09	0.08	0.27^∗∗∗^
Social Norms	0.08	0.18^∗∗∗^	0.06	0.24^∗∗∗^	0.22^∗∗∗^	–0.03	0.00	0.23^∗∗∗^	0.12^∗^	0.19^∗∗∗^
Social Image	–0.02	0.17^∗∗∗^	0.06	0.13^∗∗^	0.23^∗∗∗^	–0.01	0.10^∗^	0.13^∗∗^	0.18^∗∗∗^	0.10^∗^

*^∗^*p* < 0.05; ^∗∗^*p* < 0.01; ^∗∗∗^*p* < 0.001. Correlations in bold indicate convergent validity.*

The relationship between TEMS scales and BMI is displayed in Table 3. People with a higher BMI had significantly higher scale means for the motives ‘Convenience,’ ‘Pleasure,’ ‘Price,’ ‘Visual Appeal,’ ‘Weight Control,’ ‘Affect Regulation,’ ‘Social Norms,’ and ‘Social Image.’ In contrast, people with a lower BMI had significantly higher scale means for the motives ‘Hunger’ and ‘Health.’

## Discussion

The aim of this study was to test the factor structure of the Brazilian Portuguese TEMS (Renner et al., 2012; Moraes and Alvarenga, 2017; Sproesser et al., 2018) in confirmatory factor analysis as well as to investigate its scale reliabilities and validity. The model, including 15 motive factors and 45 items, showed a reasonable model fit. Moreover, factor loadings and corrected item-scale correlations were generally good. Only one out of the 15 motive scales had a reliability below 0.60. Convergent validity was shown as, within each FCQ scale, highest correlations were observed with the TEMS scale that taps into a similar construct. Ten out of the 15 motive scales showed significant relationships with BMI. These results demonstrate that the Brazilian Portuguese version of TEMS is generally reliable and valid to assess individual drivers of eating behavior in Brazil.

In a similar vein, the 15-factor structure of TEMS was, in general, confirmed in Germany, India, and the United States (Sproesser et al., 2018). Moreover, satisfying internal consistencies of TEMS scales were also found in Australia (Skead et al., 2018), China (Siegrist et al., 2015), and Portugal (Graça et al., 2019). Hence, despite marked differences in eating environments between these countries, the fifteen basic motives of TEMS seem to be generalizable. In line with this, motive scales of the FCQ have been replicated in European samples and a South-East Asian sample (Pieniak et al., 2009b; Januszewska et al., 2011). This provides evidence that motives of normal, that is non-pathological, eating behavior might be comparable across continents and eating cultures. This, however, does not mean that there are no differences in the mean levels of motives. In fact, large differences in the mean levels of motives occurred between samples from different countries (Januszewska et al., 2011; Sproesser et al., 2018). Similar results have been obtained for other constructs. For instance, the three subscales of the Dutch Eating Behavior Questionnaire (Van Strien et al., 1986) have been found to be valid across countries (e.g., Nagl et al., 2016; Wu et al., 2017), despite differences in the mean levels of subscales (Shloim et al., 2014).

It should be noted, however, that the 15 basic motives of TEMS emerged from qualitative studies with German samples and from an international literature review. In this international literature, however, Western samples and views are most likely overrepresented (Henrich et al., 2010). Hence, one might speculate that in other cultures, there might be further motives for eating behavior that are not yet included in TEMS. Future research is needed with non-Western samples and qualitative designs to fully determine the basic motives for human eating behavior.

In line with our results on the convergent validity of TEMS, TEMS scales also showed convergent validity in a study with US Americans (Arbit et al., 2017). For instance, the TEMS scale Sociability showed a high correlation with a scale measuring the social meaning of food (MFLQ-social subscale) and the TEMS scale Natural Concerns showed a high correlation with a scale measuring the moral meaning of food (MFLQ-moral subscale). This speaks in favor of a valid assessment of the respective eating motives in the different countries.

Interestingly, correlations between the 15 TEMS motive scales ranged from −0.26 to 0.57 in this study. Also, correlations between the motive scales of the FCQ ranged from −0.05 to 0.59 (Steptoe et al., 1995). This shows that the various motives measure distinct drivers of eating behavior and underlines that a fine-graded, differential assessment of motives is necessary to gain a comprehensive understanding of why people eat what they eat. Such a need for a differential assessment of different sub-facets has also been demonstrated within other domains, such as for impulsivity (Whiteside et al., 2005).

Assessing the reasons why people eat what they eat with TEMS offers the possibility to study individual drivers of eating behavior in Brazil. The results of our study regarding the relationship of eating because of hunger, social norms and to regulate negative affect, as well as of health and weight control considerations when choosing food with BMI are in line with previous research (Pieniak et al., 2009a; Renner et al., 2012; van Strien et al., 2016). Future research needs to investigate the causality of this relationship. Moreover, research is needed to examine whether this relationship is mediated by healthy or unhealthy eating practices. If motives have a causal impact on eating practices, they might be targeted in adaptive interventions (Collins et al., 2004). For instance, eating because of negative affect has not only shown to be related to BMI but also to be related to unhealthier eating behavior (Macht and Mueller, 2007; Konttinen et al., 2010; Sproesser et al., 2011). At the same time, we found that people who eat more when stressed compensate their more-eating by eating less in a positive situation (Sproesser et al., 2014). Hence, interventions for people with a high affect regulation motive might target people’s balance between positive and negative situations. Ehealth and MHealth approaches that run interventions via electronic channels (Collins et al., 2007) and with mobile devices (Gurman et al., 2012; Villinger et al., 2019) might be used to deliver such interventions. In any case, the multiple motives for eating call for interventions that go beyond targeting health and weight control concerns but take this multidimensionality into account instead.

It should be noted, however, that the scale Need and Hunger did not appear as a reliable scale. This finding is in line with previous results demonstrating comparable low internal consistencies (0.50 in a German sample, Renner et al., 2012; 0.46 and 0.32 in an Indian and US American sample, Sproesser et al., 2018). However, in the current study and in studies including German (Renner et al., 2012), Indian and US American (Sproesser et al., 2018), and Australian (Skead et al., 2018) samples, hunger was among the most frequent drivers of eating behavior as demonstrated by high mean scores. Hence, it appears that future research needs to measure hunger as a unique, mono-faceted motive (cf., Jackson et al., 2003) with a single item and not with a psychometric scale. Such a single item assessment has been shown to be valid in other domains, such as in the assessment of life satisfaction (e.g., Cheung and Lucas, 2014) or self-rated health (Benyamini, 2011).

### Limitations

Our sample was not representative for the Brazilian population, including a high percentage of women and educated people. However, TEMS has been shown to be invariant across gender (Renner et al., 2012). Furthermore, as for all voluntary surveys, there is a potential non-response bias. We can only speculate that our survey might have been especially attractive for people who are interested in and think much about the overall topic of eating behavior and that these are potentially people who are interested in healthy eating, diet or restrict their food intake. So the health and weight control motive in our sample might be higher than in a representative sample. In addition, some motives have been found to be more socially desirable than others (Sproesser et al., 2017). Hence, as with all self-report measures, social desirability impacts cannot be ruled out. Future research needs to address these concerns by using representative samples (e.g., Pechansky et al., 2009) and controlling for social desirability (e.g., Tangney et al., 2004). Moreover, future research with longitudinal designs is needed to investigate the test-retest reliability of TEMS as well as its predictive validity.

## Conclusion

This study shows that the Brazilian Portuguese version of TEMS is generally reliable and valid to assess motives of eating behavior in Brazil. Knowledge about these individual drivers of eating behavior might help to counteract unfavorable trends in diet and obesity rates in Brazil (Martins et al., 2013; World Health Organization [WHO], 2019). Moreover, the present study adds to the evidence that the fifteen basic motives of TEMS can consistently be found across countries and eating environments, hinting to a comparable conceptual organization of eating motives. The multiple reasons that underlie eating behavior call for health promotion programs that go beyond targeting health and weight control concerns but take the multiple motives of human eating behavior into account instead.

## Data Availability Statement

The datasets generated for this study are available on request to the corresponding author.

## Ethics Statement

The studies involving human participants were reviewed and approved by the ethics board of the Public Health School from the University of São Paulo. The patients/participants provided their written informed consent to participate in this study.

## Author Contributions

All authors made substantial contributions to the conception of the work, participated in the interpretation of the data, gave their final approval of the version to be published, and agreed to be accountable for all aspects of the work in ensuring that questions related to the accuracy or integrity of any part of the work are appropriately investigated and resolved. GS, JM, and MA collected the data. GS performed the data analyses and drafted the work, and all other authors provided critical revisions.

## Conflict of Interest

The authors declare that the research was conducted in the absence of any commercial or financial relationships that could be construed as a potential conflict of interest.

## Footnotes

1 https://www.dgps.de/fileadmin/documents/ethikrl2004.pdf; paragraphC.III
