# Supplementary material for: The Eating Motivation Survey in Brazil: Results From a Sample of the General Adult Population
Source: Front Psychol. 2019 Oct 15;10:2334. doi: 10.3389/fpsyg.2019.02334 (PMC6803482; doi:10.3389/fpsyg.2019.02334)
Supplement: Supplementary file 1 [file Table_1.DOCX]

Supplementary Material

*Table S1. English and Brazilian Portuguese items of The Eating Motivation Survey.*

| **English** | **Brazilian Portuguese** |
| --- | --- |
| I eat what I eat, … | Eu como o que eu como, ... |
| LIKING | PREFERÊNCIA |
| … because I have an appetite for it. | ... porque eu tenho vontade de comer. |
| … because it tastes good. | ... porque é gostoso. |
| … because I like it. | ... porque eu gosto. |
| HABITS | HÁBITOS |
| … because I am accustomed to eating it. | ... porque estou acostumado a comer isso. |
| … because I usually eat it. | ... porque é o que geralmente como. |
| … because I am familiar with it. | ... porque eu conheço o produto |
| NEED & HUNGER | NECESSIDADE E FOME |
| … because I need energy. | ... porque eu preciso de energia. |
| … because it is pleasantly filling. | ... porque me satisfaz a fome de forma agradável |
| … because I’m hungry. | ... porque eu tenho fome. |
| HEALTH | SAÚDE |
| … to maintain a balanced diet. | ... para manter uma alimentação equilibrada. |
| … because it is healthy. | ... porque é saudável. |
| … because it keeps me in shape (e.g. energetic, motivated). | ... porque me mantém com energia e motivação. |
| CONVENIENCE | CONVENIÊNCIA |
| … because it is quick to prepare. | ... porque é rápido de preparar. |
| … because it is convenient. | ... porque é conveniente. |
| … because it is easy to prepare. | ... porque é fácil de preparar. |
| PLEASURE | PRAZER |
| … because I enjoy it. | ... porque me dá prazer. |
| … in order to indulge myself. | ... para me dar algo realmente especial. |
| … in order to reward myself. | ... para me recompensar |
| TRADITIONAL EATING | ALIMENTAÇÃO TRADICIONAL |
| … because it belongs to certain situations. | ... porque pertence a certas situações. |
| … out of traditions (e.g. family traditions, special occasions). | ... por tradição (exemplo: tradição de família, ocasiões especiais). |
| … because I grew up with it. | ... porque eu cresci comendo assim. |
| NATURAL CONCERNS | QUESTÕES NATURAIS |
| … because it is natural. | ... porque é natural. |
| ... because it contains no harmful substances (e.g. pesticides, pollutants, antibiotics). | ... porque não contém substâncias prejudiciais (por exemplo: pesticidas, poluentes, antibióticos). |
| … because it stems from organic farming. | ... porque é orgânico. |
| SOCIABILITY | SOCIALIZAÇÃO |
| … because it is social. | ... porque faz parte de uma situação social. |
| … so that I can spend time with other people. | ... para que eu possa passar tempo com outras pessoas. |
| … because it makes social gatherings more comfortable. | ... porque os encontros sociais ficam mais agradáveis. |
| PRICE | PREÇO |
| … because it is inexpensive. | ... porque é barato. |
| … because I don’t want to spend any more money. | ... porque eu não quero gastar muito dinheiro. |
| … because it is on sale. | ... porque está em promoção. |
| VISUAL APPEAL | ATRAÇÃO VISUAL |
| … because the presentation is appealing (e.g. packaging). | ... porque a apresentação é atraente (exemplo: embalagem). |
| … because it spontaneously appeals to me (e.g. situated at eye level, appealing colors). | ... porque me chama logo a atenção (apresentação no supermercado, é colorido) |
| … because I recognize it from advertisements or have seen it on TV | ... porque eu reconheço das propagandas ou já vi na TV. |
| WEIGHT CONTROL | CONTROLE DE PESO |
| … because it is low in calories. | ... porque tem poucas calorias. |
| … because I watch my weight. | ... porque eu controlo meu peso. |
| … because it is low in fat. | ... porque tem pouca gordura. |
| AFFECT REGULATION | CONTROLE DE EMOÇÕES |
| … because I am sad. | ... porque estou triste. |
| … because I am frustrated. | ... porque estou frustado (a). |
| … because I feel lonely. | ... porque me sinto sozinho (a). |
| SOCIAL NORMS | NORMAS SOCIAIS |
| … because it would be impolite not to eat it. | ... porque seria indelicado não comer. |
| … to avoid disappointing someone who is trying to make me happy. | ... para evitar decepcionar alguém que está tentando me agradar. |
| … because I am supposed to eat it. | ... porque tenho que comer. |
| SOCIAL IMAGE | IMAGEM SOCIAL |
| … because it is trendy. | ... porque está na moda. |
| … because it makes me look good in front of others. | ... porque me faz passar um boa imagem para os outros. |
| … because others like it. | ... porque os outros gostam disso. |
